# Development and Evaluation of Biodegradable Core-Shell Microfibrous and Nanofibrous Scaffolds for Tissue Engineering Applications

**DOI:** 10.1007/s10856-024-06777-z

**Published:** 2024-01-29

**Authors:** Athina Mitropoulou, Dionysios N. Markatos, Andreas Dimopoulos, Antonia Marazioti, Constantinos-Marios Mikelis, Dimosthenis Mavrilas

**Affiliations:** 1 Department of Mechanical Engineering and Aeronautics, Laboratory of Biomechanics and Biomedical Engineering, University of Patras, Patras, GR Greece; 2 Department of Mechanical Engineering and Aeronautics, Laboratory of Technology and Strength of Materials, University of Patras, Patras, GR Greece; 3 Prometheus Division of Skeletal Tissue Engineering, KU Leuven, Leuven, Belgium; 4 Skeletal Biology and Engineering Research Center, Department of Development and Regeneration, KU Leuven, Leuven, Belgium; 5 Department of Physiotherapy, Laboratory of Basic Sciences, University of Peloponnese, Sparta, GR Greece; 6 Department of Pharmacy, Laboratory of Molecular Pharmacology University of Patras, Patras, GR Greece

## Abstract

Tissue engineering scaffolds as three-dimensional substrates may serve as ideal templates for tissue regeneration by simulating the structure of the extracellular matrix (ECM). Many biodegradable synthetic polymers, either hydrophobic, like Poly-ε-caprolactone (PCL), or hydrophilic, like Poly(Vinyl Alcohol) (PVA), are widely used as candidate bioactive materials for fabricating tissue engineering scaffolds. However, a combination of good cytocompatibility of hydrophilic polymers with good biomechanical performance of hydrophobic polymers could be beneficial for the in vivo performance of the scaffolds. In this study, we aimed to fabricate biodegradable fibrous scaffolds by combining the properties of hydrophobic PCL with those of hydrophilic PVA and evaluate their properties in comparison with pristine PCL scaffolds. Therefore, single-layered PCL scaffolds, sequential tri-layered (PVA/PCL/PVA), and core-shell (PVA as shell and PCL as core) composite scaffolds were developed utilizing the electrospinning technique. The material structural and biomechanical properties of the electrospun scaffolds, before and after their hydrolytic degradation over a seven-month period following storage in phosphate-buffered saline (PBS) at 37 °C, were comprehensively compared. In addition, human embryonic kidney cells (HEK-293) were cultured on the scaffolds to investigate potential cell attachment, infiltration, and proliferation. The results demonstrated the long-term efficacy of core-shell biodegradable fibrous scaffolds in comparison to single-layers PCL and tri-layers PVA/PCL/PVA, not only due to its superior morphological characteristics and mechanical properties, but also due to its ability to promote homogeneous cell distribution and proliferation, without any external chemical or physical stimuli.

**Graphical Abstract:**

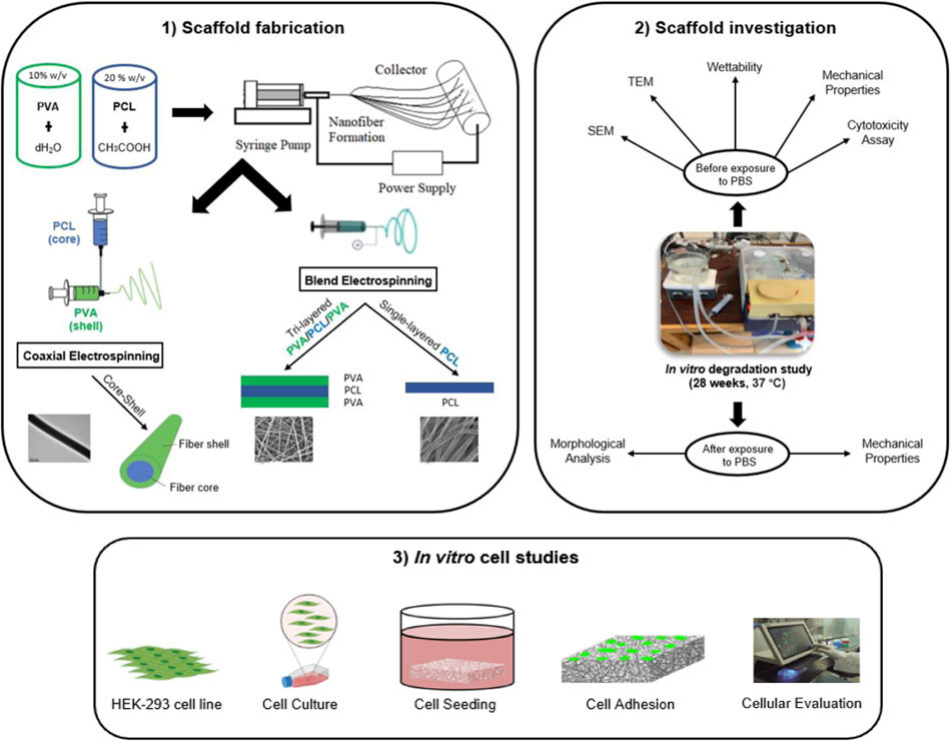

## Introduction

Tissue engineering (TE) has emerged as a distinct field at the intersection of biology, engineering, materials science, and medicine, and has become an integral part of the interdisciplinary landscape. In recent decades, biomaterial science has become an essential field in TE and regenerative medicine applications. The key issue in TE is the development of 3D scaffolds close to the natural tissue architecture. This can be achieved by fabricating micro-/nano-sized scaffolds that closely resemble extracellular matrix (ECM) topographies [1, 2]. The ideal scaffold should offer desirable architecture, adequate mechanical properties, suitable surface and internal topography, biocompatibility, biodegradability, as well as supporting cell proliferation, differentiation, and migration [3–5]. These properties and features offered by TE scaffolds may overcome the limitations of today’s artificial implants, as well of autografts and allografts, reducing patient postoperative recovery periods, and preventing the use of expensive therapies [6].

In the past three decades, several methodologies have been developed for the fabrication of nanofibrous scaffolds, including electrospinning (ESP), phase separation and self-assembly technologies. Among them, ESP has become one of the most studied fabrication techniques in tissue regeneration applications; it is a straightforward, cost-effective, and scalable technique suitable for producing interconnected and highly porous fibrous scaffolds [7–9]. ESP is a multiparametric technology for biomaterial fabrication. It is directly depended on: (1) materials’ parameters (polymer molecular weight (MW), MW distribution; (2) solution properties (e.g., viscosity, surface tension, conductivity); (3) process parameters (flow rate, applied voltage, distance between the spinneret and collector, motion of collector); (4) environmental parameters (temperature, humidity). By selection of specific parameter set one can control the morphology, the fiber diameter, and the porous architecture/structure of the scaffold to enhance the cell adhesion, attachment, proliferation, differentiation, and migration in vivo [10–12]. According to recent studies, specific characteristics of the electrospun scaffolds such as the aligned direction of the fibers, seem to improve the mechanical performance [13] or even, the precise control of the pores and the size of the fibers can allow for the controlled delivery of liposomal drugs [14]. These significant adaptable characteristics make ESP particularly appropriate for the development of biomimetic nanosized fibrous scaffolds, contributing to restore, maintain or improve tissue functions [15, 16].

Over the years various biodegradable materials have been used for the repair/replacement of tissues and many kinds of research have been conducted to evaluate the feasibility of using scaffolds made from these materials [17–19]. Biodegradation is a term used to describe the process of breakdown of a material by nature; however, in case of TE and regenerative medicine, biodegradation is focused on the biological process that occurs in vivo, causing the gradual breakdown of materials [20]. Biodegradable polymers are used to construct implantable biomaterial scaffolds, as they are intended to provide a temporary structure that initially supports physiological function and host cell migration, leading subsequently in new tissue regeneration which is mechanically strong enough to withstand applied physiological forces without the assistance of foreign material [21–24]. Their application in biomedicine has gradually increased, with proven value in the reconstruction of multi-tissue organs, tissue interfaces and even structural tissues, including bone, cartilage, tendons, ligaments, muscle, and even fibrous constructs for neural, heart and vascular systems [25–30].

The most common materials applied in the production of electrospun fibers are natural or synthetic polymers [31]. Naturally derived biomaterials have good biodegradability and biocompatibility and make cell adhesion and proliferation easier. However, there are still some problems in their usage, such as poor mechanical properties, fast degradation rate, etc. [32]. In contrast, synthetic biomaterials can provide better mechanical properties and a more adjustable biodegradation rate, to leave enough space for cells to regenerate proper tissues and organs [33]. In most processes based on synthetic scaffolds, besides biomimicking of surface and mechanical properties, additional post-processing steps such as external stimulation or addition of growth factors are often required for optimal cell-scaffold interaction towards biocompatibility, mechanical stability, and equalization of rates of biodegradation and new tissue formation [34]. The selection of the appropriate single or multi polymeric material to be electrospun into fibrous scaffolds, is one of the strategies to address this challenge, taking advantage of some of the physical characteristics of individual polymers while gaining some new functionality [35].

Among different attempts to fabricate polymeric scaffolds by ESP, the combination of hydrophobic and hydrophilic polymers is extensively used. However, the construction of composite materials either as layer-by-layer, or even coaxial structures of hydrophilic-hydrophobic polymer fibers is far from straightforward. The shell/coating layer plays a critical role in the design of fibrous scaffolds, influencing cell adhesion and biomolecules/drugs release strategy. Two general design approaches have been reported. In the first approach, a hydrophobic polymer is preferred as the (external) shell/coating material due to its slower degradation and/or corrosion, achieving sustained and prolonged release rate of drugs or bioactive molecules incorporated. However, hydrophobic polymers have been associated with low cell affinity, a disadvantage in TE applications. Thus, balancing degradation rate and cell affinity is usually achieved through hydrophobic/hydrophilic polymer blends [36–39]. In the second design approach, hydrophilic polymer is favored as (external) shell/coating material, with the hydrophobic polymer placed in internal 3D scaffold structure. This design approach is chosen to maximize cell–fiber adhesion due to better hydrophilicity and wettability of the shell/coating layer [40].

In this study, our main goal is to employ this technique that utilizes both hydrophilic and hydrophobic polymers to fabricate fibrous scaffolds. Specifically, Poly(Vinyl Alcohol) (PVA) is chosen as the external material to impart hydrophilic properties to the scaffold [41], while Poly-ε-caprolactone (PCL) is selected as the internal material to ensure a sturdy structure and enhanced mechanical support during degradation [42]. To conduct the study, three different electrospun fibrous scaffolds were fabricated, including (1) pristine PCL structure as single-layer scaffolds, served as a reference structure, (2) consecutive tri-layer composite scaffolds structured as PVA/PCL/PVA, and (3) core-shell coaxial scaffolds (PVA as shell and PCL as core). The scaffolds underwent comprehensive characterization, encompassing morphological analysis, surface assessment, evaluation of mechanical and cell-contact biological properties. Additionally, a seven-month in vitro degradation study was conducted on the electrospun scaffolds to assess the potential impact on material properties and its implications for their suitability in TE applications.

Ultimately, the novelty of this study resides in the amalgamation of these two polymers within the scaffold, manifesting as both individual fibers and mixed coaxial fibers, aiming to synergize their individual advantageous characteristics, including mechanical strength, ductility, and bioactivity. This innovative combination is designed to promote enhanced cell adhesion and proliferation by augmenting hydrophilicity and surface cell-recognition sites, thus offering unique properties beneficial for advanced tissue engineering applications.

## Materials and methods

### Materials and Solution Preparation

PCL in the form of pellets with an average MW 80,000, and PVA with an average MW 85,000–124,000 (87–89% hydrolyzed) in the form of crystals were purchased from Sigma-Aldrich, UK. Glacial acetic acid (≥99.8%, Honeywell Fluka) and 2-distilled water were used as solvents.

PCL working solution was prepared by dissolving 20% *w/v* PCL pellets in glacial acetic acid. The solution was transferred in 20-ml cylindrical plastic vials and stirred with a laboratory roller mixer for 24 hours under 40 °C to obtain a transparent solution. The solution was subsequently left to cool at room temperature (RT; ∼23 °C) and used within 2 days.

PVA water solution was prepared by dissolving 10% *w/v* PVA crystals in 2-distilled water at 90 °C to a closed container under stirring for 3 hours. Afterwards, the solution was left to be cooled at room temperature (RT; ∼23 °C) and stayed overnight under constant stirring.

**Fig. 1:**
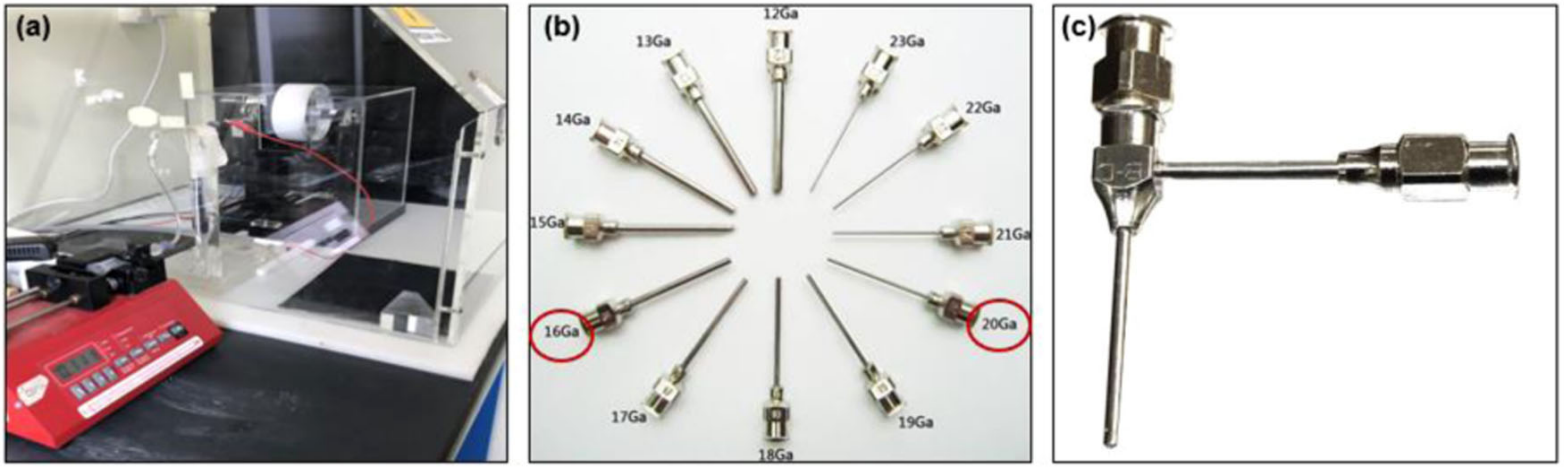
**a** Laboratory set up of the electrospinning technique **b** Different size needles from stainless steel to produce the micro-nano fibers (in red circles are the needles used in this study during blend electrospinning) and **c** Coaxial stainless-steel needle to produce core-shell fibers

Regarding the fabrication of the electrospun scaffolds in this study, an in-house electrospinning setup was used (Fig. 1a), the detailed description of which has been published earlier [13, 14, 28]. Two type of stainless steel metallic blunt needles were used: Simple industrial needles of different sizes for the single- and tri-leaflet scaffolds (Fig. 1b), or specially designed needles, used for coaxial ESP, to produce core-shell scaffolds (Fig. 1c). A schematic diagram of the forming electrospun process of the scaffolds is presented within the graphical abstract.

### Fabrication of single-layered scaffolds

The single-layered PCL scaffolds were fabricated via blend electrospinning. For this, a 10-ml syringe with a plastic tube extension and a stainless-steel blunt needle (20 Gauge) was filled with PCL working solution, driven by a syringe pump (New Era Pump Systems Inc. NE-1000) at a feed rate of 2 ml/h. Subsequently, a high DC voltage of 20 kV (Spellman SL300 DC power supply unit) was applied between the metallic needle and a rotating aluminum cylindrical drum (width 5 cm and diameter 10 cm) collector surface (earth), placed at 22 cm distance. A specifically designed and constructed Arduino-driven (Arduino Mega 2560 Rev3 with motor shield Rev3) was used to accurately control the angular velocity of the drum [13]. The electrospinning processes were performed at room conditions (RT; ∼23 °C, ∼50% humidity). After conducting preliminary experiments, a rotational drum speed of 1300 rpm was found to produce better fiber quality and alignment on the cylindrical surface. The collector’s surface was covered with aluminum foil to collect and easily detach the electrospun membranous scaffold. The electrospinning process duration was 3 hours.

### Fabrication of tri-layered scaffolds

To construct the tri-layered PVA/PCL/PVA scaffolds, a blend electrospinning technique was employed. First, the PVA external layer was electrospun, followed by the central PCL layer, and then another external layer of PVA was added. The PVA outer layers were created using a matching spinning setup. A 10-ml syringe with a plastic tube extension and a 20 G (0.9 mm, inner diameter) stainless steel blunt needle was filled with the PVA solution and driven by the syringe pump, at a feeding rate of 0.5 ml/h. A high DC voltage of 20 kV was applied between the metallic needle (+) and the metallic collector surface (earth) at 14 cm distance. The same rotating metallic drum was used as the earthed collector plate. After preliminary experiments, to achieve better fiber quality and alignment on the cylindrical surface, a rotational drum speed of 1500 rpm was selected for the electrospinning process of PVA. The electrospinning processes were performed at room conditions (RT; ∼23 °C, humidity ∼50%). The PCL internal layer was electrospun as described in section 2.2. The whole blend electrospinning process duration was 6 hours.

### Fabrication of core-shell scaffolds

Core-shell scaffolds were fabricated via a coaxial electrospinning process. In the concentric spinner, the shell liquid of PVA solution and the liquid core of PCL were supplied by two different syringe pumps (New Era Pump Systems Inc. NE-1000) to drive the fluids independently; the inner and outer precursor working solutions, each from a 10-mL syringe, inserted to the separated inputs of the coaxial needle, driven to the nozzle through their respective capillaries and formed composite droplets. The coaxial fibers were obtained using a specific coaxial needle: a central one with 0.63/0.91 mm inner/outer diameter (20 Gauge) and a peripheral 1.26/1.66 mm inner/outer diameter (16 Gauge). The shell syringe pump supplied a steady flow of 0.5 mL/h of the shell solution (PVA) while the core syringe pump fed a consistent flow of 2 mL/h of the core solution (PCL) to the corresponding tips of the needle. The same cylindrical drum, rotated at a speed of 1260 rpm, was placed at different distances from the needle tip to check the drying of the fibers before hitting the collector. A rectangular piece of aluminum foil (5 cm width and 10 cm diameter) was covered on the drum to collect the electrospun micro- and nano-fibers. After some initial optimization experiments, a set of the electrostatic field at 20 kV (Spellman SL300 DC power supply unit) and a distance 16 cm of the collector from the needle tip was chosen. The electrospinning processes were performed at room conditions (RT; ∼23 °C, humidity ∼50%). Coaxial electrospinning process was performed for 6 hours in total.

### Assessments of scaffolding materials

The material evaluation of scaffolds involves examining both the surface and internal morphology, quantifying their hydrophobicity/hydrophilicity, determining their in vitro degradation under various environmental conditions, and conducting mechanical testing prior to and during the degradation period.

#### Morphological studies

The morphology of the electrospun fibrous scaffolds was observed by Scanning Electron Microscope (SEM) (JEOL 6300). Rectangular-shaped specimens were gold-sputtered prior to SEM observation. Based on the SEM images, fiber alignment was assessed and the fiber diameter, as well as the pore size (distance between the fibers), was measured using an image processing software (Image J, version 1.51n, National Institutes of Health, USA). In addition, the core-shell structure of the electrospun fibers was demonstrated by use of Transmission Electron Microscope (TEM) (JEM-2100, JEOL). The samples for the TEM observation were prepared by directly depositing the coaxial fibers onto the copper mesh coated with carbonic film.

#### Contact angle measurements

To determine the wettability of the scaffolds and possible alterations associated with the electrospinning procedure and fiber morphology and orientation, contact angle (C.A.) measurements (sessile drop method) were performed at room temperature using a contact angle meter (CAM 101, KSV instruments Ltd.). The test liquid used was phosphate buffer solution (PBS, pH 7.4). Images of droplets on the freeze-dried scaffolds’ surface were captured immediately after liquid contact. Six (6) specimens 10 × 10 mm from each group of scaffolds were tested.

#### In vitro degradation assessment

For the degradation study, samples (n = 3) were cut from the fiber sheet collected from the spinning drum into rectangular specimens 10 × 10 mm, freeze-dried and weighed one by one on a precision balance (Mettler Toledo AB204-S), and then subsequently placed in sterile 2-mL microfuge tubes (Thermo Fisher, UK) containing 2 mL PBS (pH 7.4). All tubes were covered with a parafilm layer (Sigma-Aldrich, UK) and held upright within plastic tube racks of appropriate size. Racks were subsequently housed within an automated rotary shaker chamber (100 rpm, 37 °C). The degradation study was performed over a 7-month period, with time points at 1, 6, 12 and 28 weeks. At each time point, samples were removed using forceps and measurements of water uptake and weight loss measurements were carried out: At the end of each time point, the weight of the swollen samples was measured after removing the excess surface water with filter paper. Water uptake percentage of the scaffolds was determined from the weight of wet sample (W_w_) and the initial weight of freeze-dried sample (W_i_) using the following Eq. (1). The reported water uptake was considered as the average value of three samples from each group of electrospun scaffolds.

1 $${\rm{Water}}\,{\rm{uptake}}( \% )=\frac{({\rm{Ww}}-{\rm{Wi}})}{{\rm{Wi}}}\times 100$$

The weight loss of the scaffolds was determined from the initial weight of freeze-dried (W_i_) and weight of final freeze-dried (W_f_) sample at end of each time point using the following equation:

2 $${\rm{Weight}}\,{\rm{loss}}( \% )=\frac{({\rm{Wi}}-{\rm{Wf}})}{{\rm{Wi}}}\times 100$$

The degradation process in PBS can impact many aspects of polymeric scaffolds. The alkaline saline solution may provide helpful information about how these biodegradable scaffolds could perform in vivo in terms of their morphology, fiber diameter and pore size changes. To investigate these scaffolds’ structural behavior changes under degradation process conditions, SEM imaging of electrospun fibers was performed.

#### Mechanical characterization

The mechanical properties of the electrospun fibrous scaffolds were measured with a bench type miniature tensile tester (MiniMat 2000, Rheometric Scientific Inc.) equipped with a 200 N load cell, at a constant rate of 10 mm/min until failure at room conditions (RT; ∼23 °C, humidity ∼50%). Rectangular specimens 20 × 5 mm were cut (longitudinally) along the directional axis of the fibers, i.e., parallel to the perimeter of the drum. Five (5) samples from each group of scaffolds were mechanically tested before and after their in vitro exposure to phosphate buffer solution (PBS, pH 7.4). According to the procedure followed in section 2.5.3, the degraded samples for each group of scaffolds were carefully selected from plastic tubes containing 5 mL PBS (pH 7.4). Each plastic tube contained five (5) rectangular specimens (20 × 5 mm) from each group of scaffolds and each one represented a different time point of degradation (1, 6, 12 and 28 weeks). The degraded samples were subjected to mechanical tests after drying at room temperature (RT; ∼23 °C) for 24 hours. Stress-strain curves were obtained for each sample and the Young’s modulus, ultimate tensile strength, and maximum strain at failure (%) were calculated before (time zero – week 0) and at each time point of biodegradation (1, 6, 12 and 28 weeks).

### In vitro biological evaluation of scaffolds

#### Cell culture

In this study, we used the human embryonic kidney cell line HEK-293 as our cell model. Derived from human embryonic kidney tissue, these cells exhibit robust growth characteristics, ease of culture, and ability to express exogenous genes, making them suitable for a wide range of experimental purposes [43, 44]. HEK-293 human embryonic kidney cell line was purchased from the American Type Culture Collection (Manassas, VA, USA). The cells were cultured in Dulbecco Modified Eagle’s Medium (DMEM, Sigma-Aldrich, France) supplemented with L-glutamine, 4500 mg/L glucose, 110 mg/L sodium pyruvate, 10% fetal bovine serum (FBS), 50 U/mL penicillin and 50 mg/mL streptomycin. They were incubated at 37 °C in a humidified atmosphere of 5% CO_2_.

#### Biocompatibility test

MTT (3-(4,5-Dimethylthiazol-2-yl)-2,5-diphenyltetrazolium bromide) cell viability assay method was used to evaluate the biocompatibility of the tissue engineering scaffolding materials. The method is based on the absorbance of the dissolved MTT formazan crystals formed in living cells, which is proportional to the number of viable cells. The electrospun scaffold specimens, after sterilization with 75% ethanol for 6 hours and exposure to ultraviolet light (UV) for 30 min, were put in empty Polystyrene (PS) 24 well plates and washed once with phosphate-buffered saline (PBS) and once with DMEM full medium. Cells were next dropwise seeded at a density of 2 × 10^4^ cells/well in 200 μl growth medium. Once the cells were seeded, they were placed in the 37 °C incubator and allowed to attach for 2 hours. The scaffolds were then covered with 0.5 ml cell growth medium. Specifically, cells were seeded and allowed to attach on well plates of PS (control, *n* = 4) and onto scaffolds (treatment, *n* = 4) from each electrospun group (single-layered, tri-layered, core-shell scaffolds) for 24 and 48 hours. After incubation, a calculation of cell viability was followed. In each well, we added 20 μl of the MTT solution (5 mg/ml) in a final volume of 200 μl PBS and incubated for 4 hours (to form formazan crystals). Metabolically active cells reacted with tetrazolium salt in the MTT reagent to produce a soluble formazan dye. Formazan crystals were solubilized by the addition of 200 μl of acidified isopropanol (0.04 N HCl in isopropanol) followed by stirring. Absorbance (A) measurement was done at 570 nm (wavelength) on a Multiscan EX plate reader (Micro Plate Reader, MK3, Thermo Lab Systems, USA). The percentage of viable cells was then calculated based on the following formula (3):

3 $${\rm{Cell}}\,{\rm{viability}}( \% )=\frac{({\rm{A}}570\,{\rm{sample}}-{\rm{A}}570\,{\rm{background}})}{({\rm{A}}570\,{\rm{control}}-{\rm{A}}570\,{\rm{background}})}\times 100$$

where A_570_ background was the optical density (OD) at 570 nm of MTT without cells.

#### Assessment of cell morphology and proliferation

To investigate morphology, attachment, and distribution of the cultured ΗΕΚ-293 cells on the scaffolds, an inverted optical microscope (Nikon Diaphot, Tokyo, Japan), a Scanning Electron Microscope (SEM) (JEOL 6300), and a ZOE fluorescent cell imager (Biorad) were used.

Circular scaffolds (0.9 mm) were introduced into wells of 24 well plates and sterilized by Ultraviolet (UV) radiation for 20 min before being washed once with phosphate-buffered saline (PBS) and once with DMEM full medium. Cells were next dropwise seeded at a density of 10^4^ cells/well in 200 μL growth medium. Once the cells were seeded, they were placed in the 37 °C incubator and allowed to attach for 2 hours. The scaffolds were then covered with 0.5 mL cell growth medium and incubated for 1, 3 and 7 days.

The observation of adhered cells on the surfaces of fibers was evaluated by SEM on the 7th day of the culture. The fibrous scaffolds were washed twice with PBS and HEK-293 cells and fixed with 2.5% (*v/v*) glutaraldehyde in 0.1 M PBS (pH 7.4) for 30 min at ambient conditions. The samples were washed again twice with 0.1 M PBS (pH 7.4) for the removal of unreacted glutaraldehyde. The cells were dehydrated with varying concentrations of ethanol (30, 50, 70, 90, and 100%) (Sigma-Aldrich, France) for 5 min in each solution. Finally, the cells cultured on the scaffolds were sputter coated with gold (thickness 3 nm), after drying with Hexamethyldisilazane (HMDS) (Sigma-Aldrich, France).

Cells were also visualized via fluorescence microscopy after 1, 3 and 7 days of growth. Plates were removed from the incubator and rinsed three times in PBS to remove excess media. The cells were fixed using 4% (*v/v*) paraformaldehyde (Electron Microscopy Sciences), permeabilized using 0.1% Triton-X 100 (Sigma-Aldrich), and then immuno-stained with 4΄,6-diamidino-2-phenylindole (DAPI) (Life Technologies, USA) and Alexa Fluor® 488 Phalloidin (Life Technologies, USA) for A-T regions of the nucleus and cytoskeleton (F-Actin) visualization respectively, according to the manufacturer’s protocols. Fluorescence microscopy analysis of the cultured and stained cells within scaffolds was carried out using ZOE fluorescent cell imager (Biorad).

### Statistical analysis

All data are reported as the mean ± standard deviation (SD) of n samples for each experimental group. Group comparisons were conducted using one-way analysis of variance (ANOVA) to assess statistical significance. Differences between groups were considered significant when *p* < 0.05. The statistical analyses were performed using SPSS Statistical Software Package (Version 25.0, IBM Corp., Armonk, NY).

## Results

### Electrospun scaffolds morphology

**Fig. 2:**
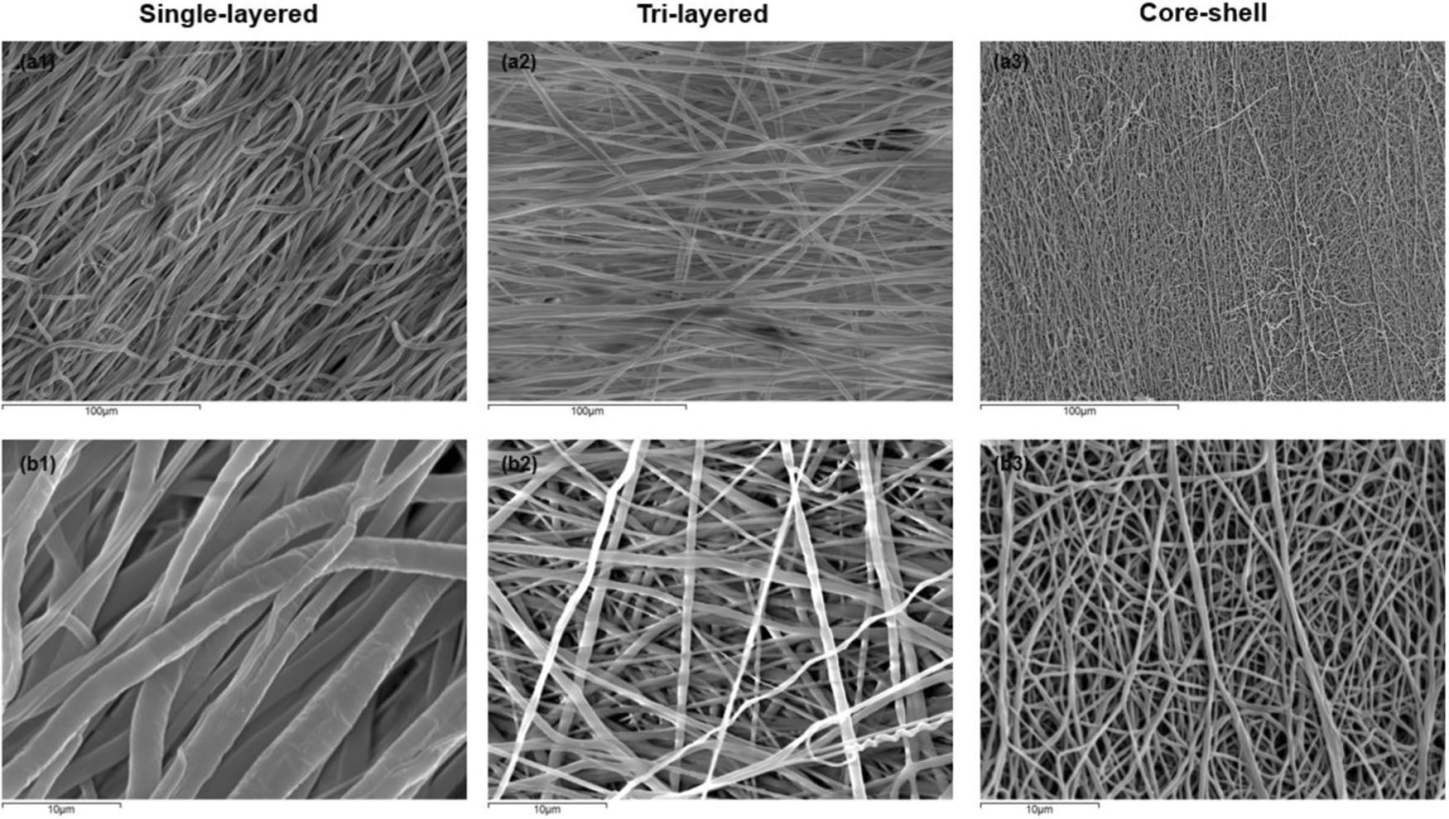
Scanning electron microscopy micrographs of electrospun fibers: Images **a1** Single-layered fibers **a2** Tri-layered fibers and **a3** Core-shell fibers (scale bar = 100 μm). Images **b1,** **b2** and **b3** respectively shows a magnified view of the scaffolds (scale bar = 10 μm)

Figure 2 displays representative SEM images of fiber morphology of the scaffolds fabricated in this study via blend single-layered (Images a1, b1), tri-layered (a2, b2) and coaxial core-shell (a3, b3) electrospinning.

It seems that under the specific set of electrospinning parameters (voltage, feed rate, needle to collector distance, rotary collector angular velocity) and environmental conditions (room temperature, humidity) mentioned at part 2.2–2.4 we obtained a stable electrospinning process, with very homogeneous size, distribution, and structure of fibers. The tri-layered PVA/PCL/PVA scaffold material is shown to be arranged as microfibers (fiber diameter = 1.014 ± 0.419 μm, mean ± SD, *n* = 50) entangled with nanofibers (average fiber diameter = 0.086 ± 0.12 μm, mean ± SD, *n* = 50) forming a layer-by-layer region with great pore interconnectivity. These results are justified due to the difference in the speed of the rotating collector (1300 rpm and 1500 rpm for PCL and PVA respectively) during the sequential blend electrospinning process, in which a reduction in the average diameter of the fiber is observed. As per [13, 45] the diameter of the electrospun fibers is inversely proportional to the speed of rotation.

**Fig. 3:**
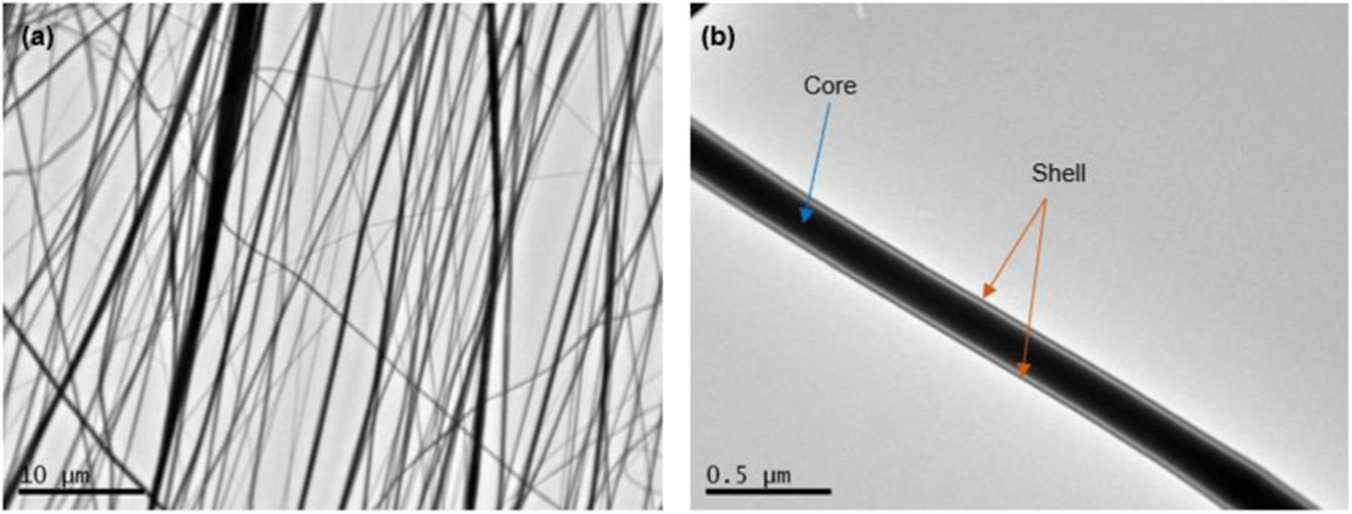
Representative TEM images of coaxial electrospun fibers **a** (scale bar = 10 μm). Core-shell configuration is shown detailed in **b** (scale bar = 0.5 μm)

For a detailed study of the core-shell scaffold structure, TEM was utilized. TEM images (Fig. 3) of the fibers of core-shell scaffolds revealed a coaxial fiber composed of a thick core (PCL) and a relatively thin shell (PVA). It seems that core-shell scaffold material is organized in aligned coaxial micro- and nano-fibers (Fig. 3a). A clear core-shell structure was observed in which the different layers of the coaxial fiber are clearly distinguished, where the color of the shell is lighter than that of the core (Fig. 3b).

During coaxial electrospinning, it was observed that the fiber diameter exhibited a decrease over time, showing variations between the initial (inner towards drum) and late (outer) sections. The decrease followed a normal distribution pattern. Indicatively, the average total diameter of the coaxial nanofibers was found to be 0.398 ± 0.986 μm, while the core diameter averaged at 0.149 ± 0.146 μm, accounting for approximately 37.4% of the total diameter (mean ± SD, *n* = 50). Also, the fiber diameters of the core and shell layers were uniform, indicating that the coaxial electrospinning process has excellent stability. The variations in the solubility of the polymers in their respective solvents, coupled with differences in polarity, played a crucial role in achieving core-shell fibers with a parallel aligned morphology.

Table 1 shows for all the three group of scaffolds the mean fiber diameter, the pore size, as measured by Image J software (NIH), as well as the thickness of the scaffolds measured at different points, using a high-precision micrometer (mean ± SD, *n* = 50).

**Table 1:** The values of average fiber diameter, pore size and thickness of the scaffolds (mean ± SD, *n* = 50 for all measurements)

Scaffold type	Fiber diameter (μm)	Pore size (μm)	Thickness (mm)
Single-layered	1.761 ± 0.437	8.729 ± 2.281	0.14 ± 0.01
Tri-layered	1.014 ± 0.419	10.109 ± 1.499	0.11 ± 0.02
Core-shell	0.398 ± 0.986	6.921 ± 1.366	0.09 ± 0.01

While the present study opted for a qualitative depiction of fiber alignment, future research endeavors could explore more advanced quantitative methodologies to provide a more comprehensive understanding of the intricacies of fiber alignments in tissue engineering scaffolds.

### Wettability of the scaffolds

The wettability of the fabricated scaffolds was assessed using the contact angle (C.A.) method. The water C.A. measurement was used to estimate the degree of hydrophobicity or hydrophilicity of the surface of electrospun materials, which is also an essential parameter in material-cell interaction.

**Fig. 4:**
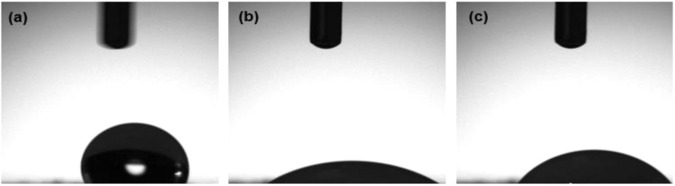
Water contact angle measurements of electrospun scaffolds **a** Single-layered scaffold **b** tri-layered scaffold and **c** core-shell scaffold (mean ± SD, *n* = 6)

The results of the C.A. measurements (Fig. 4) showed that the surface of the freeze-dried single-layered scaffolds was very hydrophobic, having an average contact angle of 119^o^ ± 2^o^ (*n* = 6) (Fig. 4a). That correlates well with the fact that PCL is known to be a highly hydrophobic material, mainly related to its content of methyl group [46], a property remained unaffected after the electrospinning process.

The tri-layered and core-shell structures of nanofiber mats were found to have significant improvement in the hydrophilic properties compared to PCL alone. The result showed that when PCL was taken as an intermediate layer at PVA/PCL/PVA tri-layered scaffolds, the outer hydrophilic PVA layer absorbed the water droplet and the angle steeply dragged down to 21° ± 3° (*n* = 6) (Fig. 4b). For the core-shell scaffolds (Fig. 4c), the great nanofibers’ interconnectivity of thin PVA shell provided channels for the water droplet to pass through and get absorbed resulting in a contact angle of 43° ± 2° (*n* = 6).

### In vitro degradation results

**Fig. 5:**
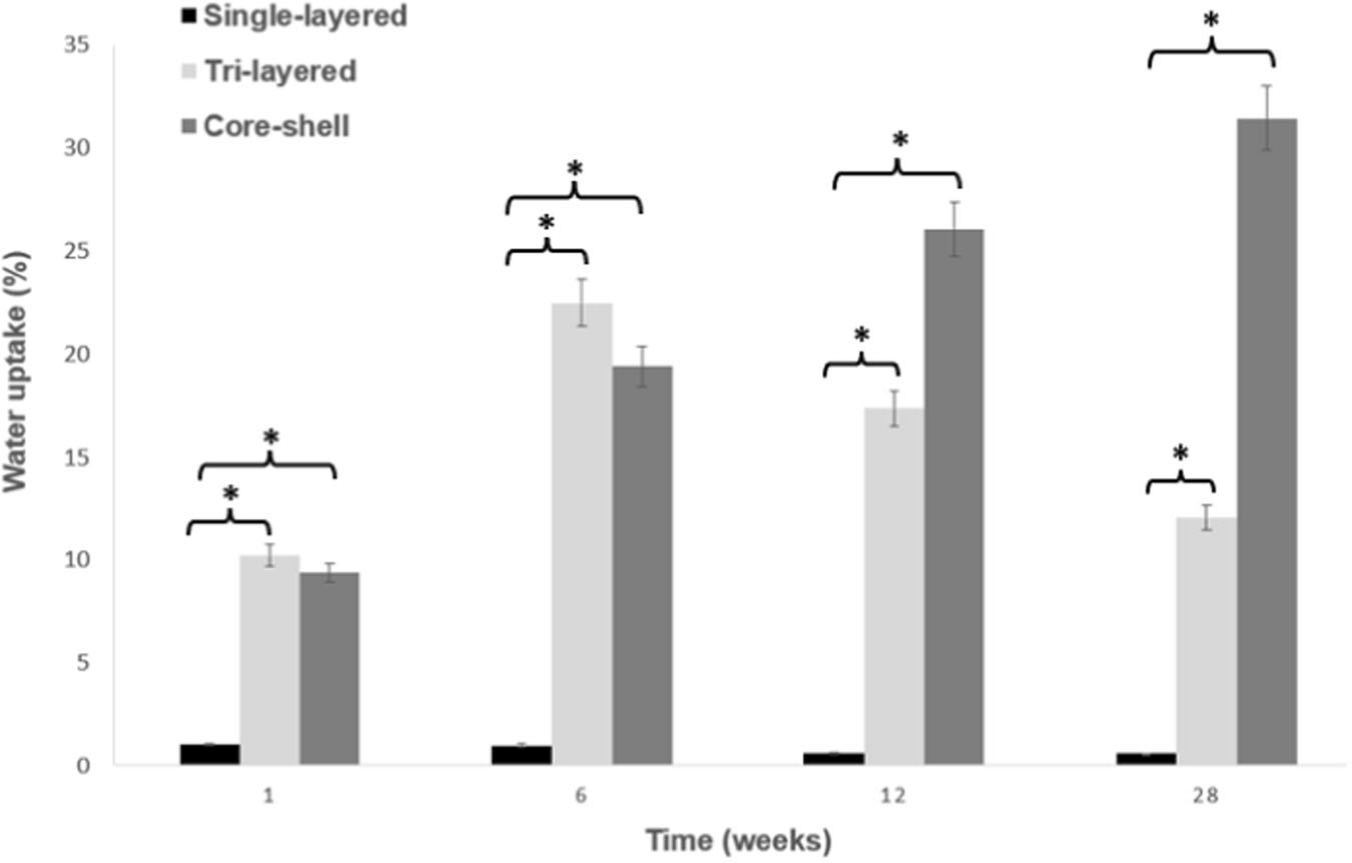
Water uptake percentage (%) for the scaffolds over a 28-week period. (mean ± SD, *n* = 3), *p < 0.05. Stars (*) represent statistical significance compared to single-layered scaffolds for the respective time points of 1, 6, 12, and 28 weeks

**Fig. 6:**
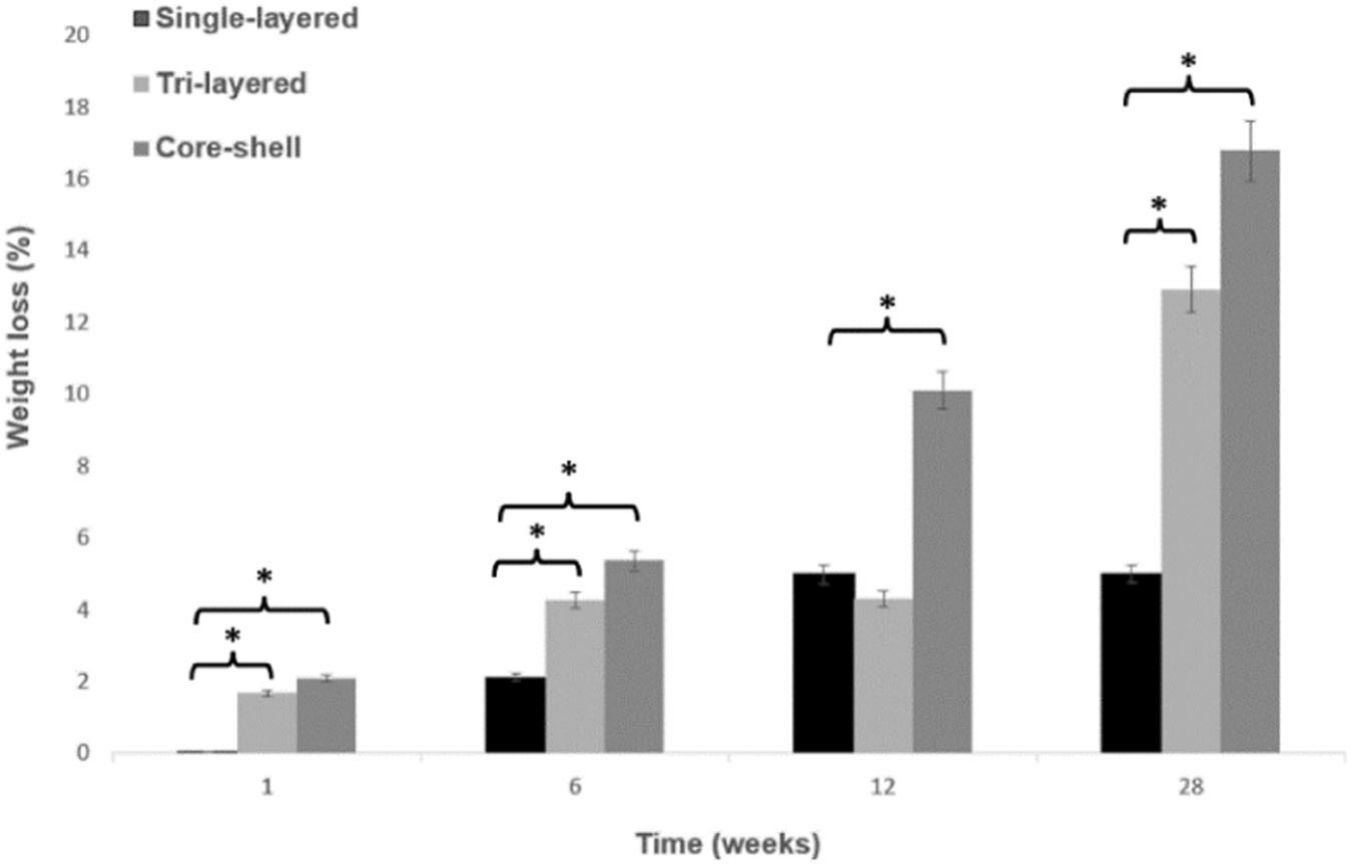
Weight loss percentage (%) for the scaffolds over a 28-week period. (mean ± SD, *n* = 3), *p < 0.05. Stars (*) represent statistical significance compared to single-layered scaffolds for the respective time points of 1, 6, 12, and 28 weeks

Temporary scaffolds needed to have a consistent degradation rate for tissue growth, yet each of these scaffolds exhibited different degradation characteristics over the 28 weeks of the degradation period. The water (PBS) uptake, the weight loss and the changes in the morphology were investigated. Figures 5 and 6 show bar charts of water uptake percentage (%) and weight loss percentage (%) for the electrospun scaffolds, calculated according to Eqs. (1) and (2) respectively, after 1, 6, 12 and 28 weeks under temperature-controlled conditions (37 °C). According to these, we derive information about the rate of degradation of the scaffolds.

As it can be observed from Fig. 5, after immersing the scaffolds in PBS, the hydrophobic nature of PCL makes the single-layered scaffolds almost impermeable, showed by a low level of water uptake with a percentage that did not exceed 2% over a period of 28 weeks. However, when PCL is used together with the hydrophilic PVA either in tri-layered or in core-shell scaffolds, the water uptake significantly increases (10% to 30%) for all the period of 28 days compared to that of PCL alone. A drop in the tri-layered scaffold after week 6 may be attributed to early partial degradation of PVA layer.

Accelerated in vitro degradation weight loss tests (Fig. 6) also showed that the hydrophobic nature of PCL did not lead to significant weight loss (less than 5%) up to 28 weeks. On the other hand, tri-layered and core-shell scaffolds gradually showed a significantly higher weight loss with time. The degradation for tri-layered scaffold started at a weight loss rate of 1.67% (1-week) and appeared to stabilize between the 6-weeks (4.28%) and the 12-weeks (4.31%), showing a sharp increase in weight loss rate at 28-weeks (12.93%). The weight loss of the core-shell scaffolds compared to the tri-layered ones appeared to occur more smoothly and gradually, at 2.09%, 5.36%, 10.11% and 16.78% for the 1, 6, 12 and 28 weeks respectively.

**Fig. 7:**
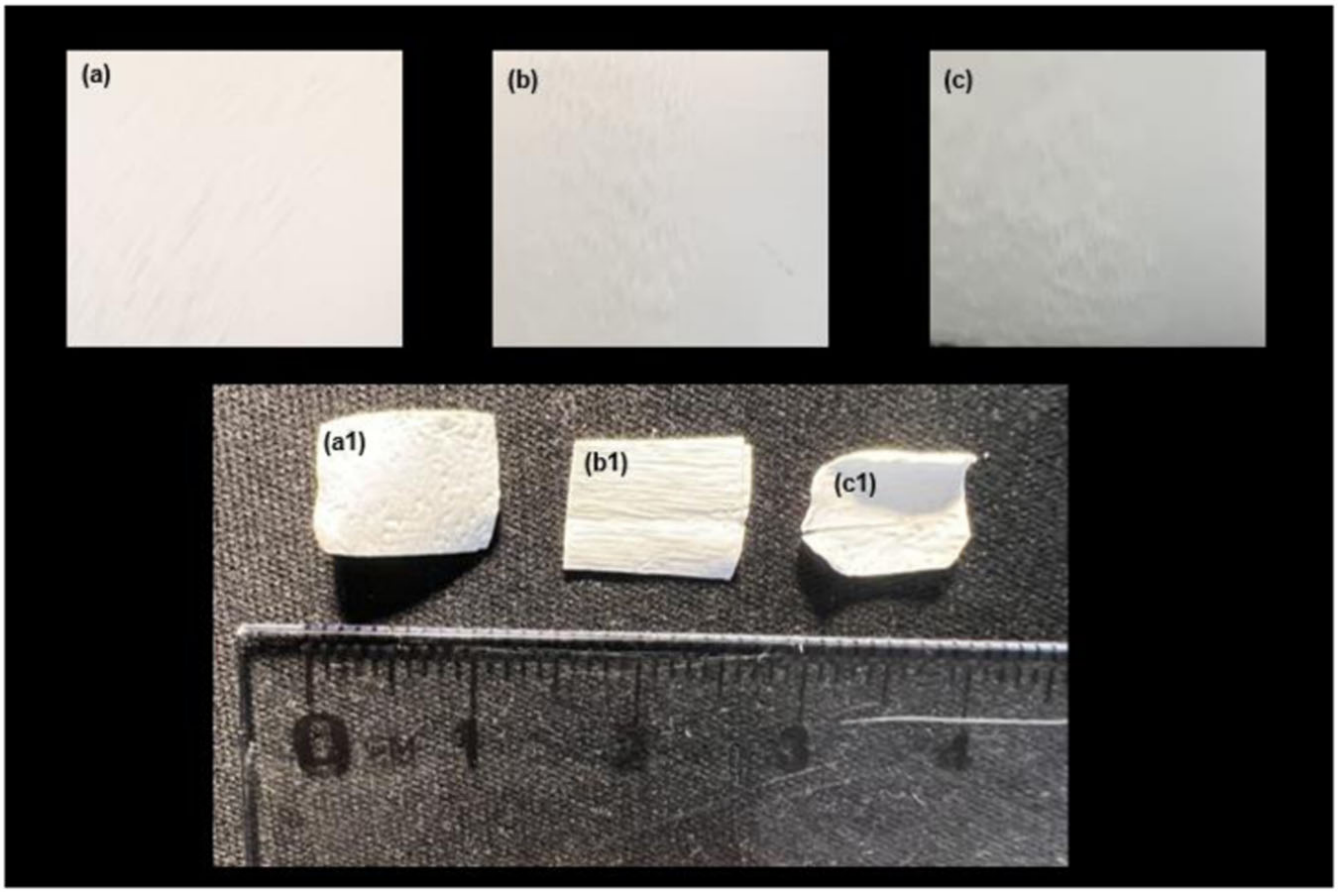
Macroscopic images of the surface morphology of the electrospun scaffolds **a** single-layered scaffold **b** tri-layered scaffold **c** core-shell scaffold at 1^st^ week and **a1,** **b1** and **c1** images of the corresponding electrospun scaffolds at 28^th^ week of insertion into PBS

Figure 7 shows a macroscopic view of the surface morphology of the scaffolds at the 1st week (images a, b, and c) and after 28 weeks (images a1, b1 and c1) of insertion into the PBS. It can be observed that the degradation process has affected the surfaces of all polymeric scaffolds. However, it seems that all scaffolds strongly retained external membranous surface morphology after the degradation process.

**Fig. 8:**
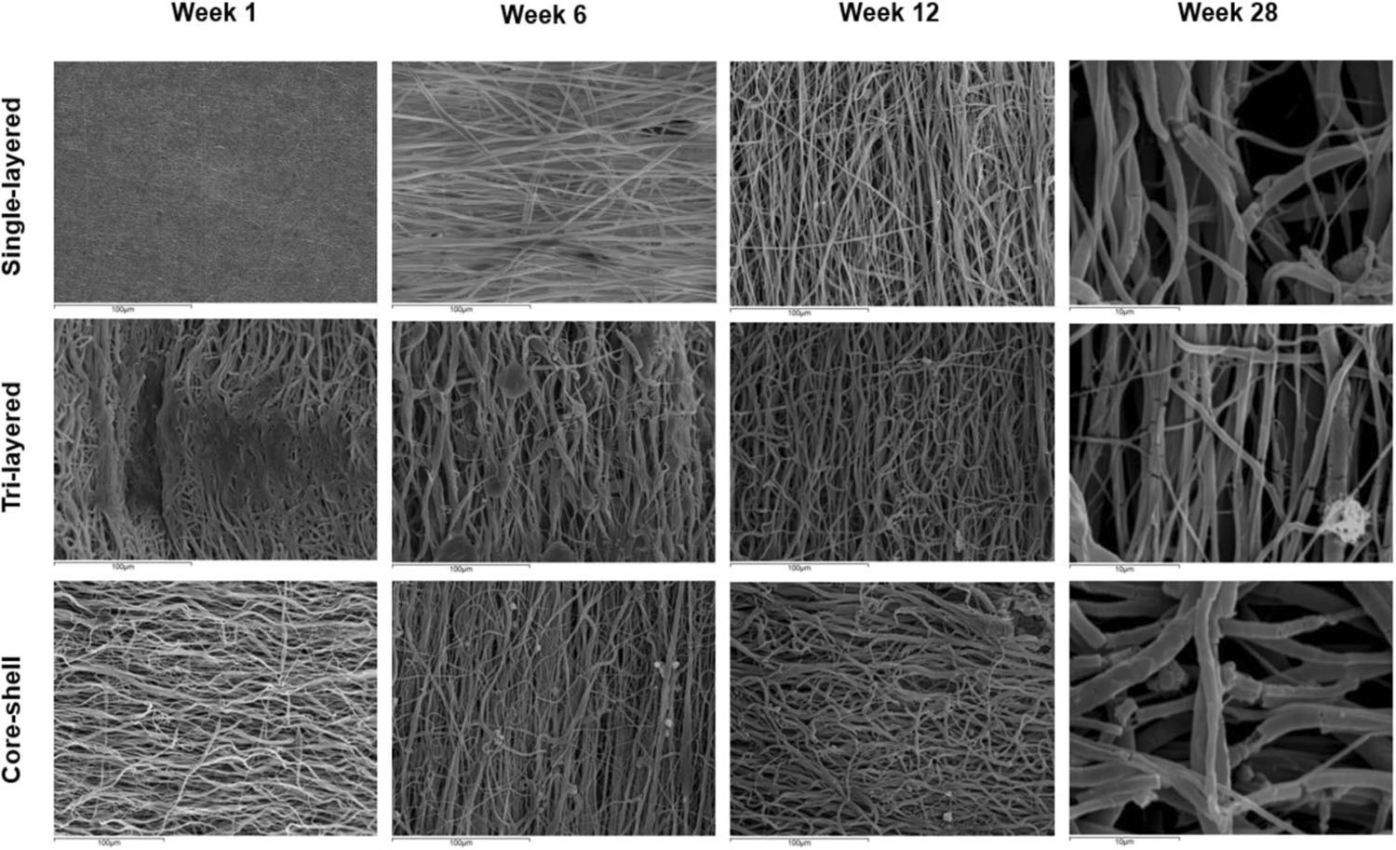
SEM images of electrospun scaffolds from weeks 1, 6, 12 and 28 (the degradation period). For 1, 6 and 12 weeks the scale bar is at 100 μm, and for the 28 weeks is at 10 μm, for better clarity in the visualization of fiber degradation and the scaffolds’ changes regarding structural behavior

Figure 8 shows SEM micrographs of the changes in fiber morphology for all scaffold types under hydrolytic degradation conditions using PBS solution at 37 °C after 1, 6, 12 and 28 weeks of exposure. After 1 week in PBS, only the single-layered PCL electrospun scaffolds retained their normal electrospun fibrous shape with most of fibers presenting smooth surfaces. Conversely, many fibers of the tri-layered scaffolds had a roughened appearance along their surface (composed of PVA), while the fibers of the core-shell scaffolds show a thickening pattern of their fiber diameters.

Further image analysis with ImageJ software’s was carried out. The average fiber diameter, as well as average pore size for both degraded (at 28-week) and time zero scaffolds were measured and compared, as shown in Table 2 (initial values can be comparatively shown in Table 1).

**Table 2:** Percentage changes in fiber diameter and pore size of scaffolds at week 28 of degradation under temperature-controlled conditions (37 °C) compared to time zero (week 0) (Table 1 values). (+) = increase and (−) = decrease, (mean ± SD, *n* = 50)

Scaffold type	(%) change in fiber diameter	(%) change in pore size
Single-layered	8.09 (+)	16.14 (−)
Tri-layered	46.18 (+)	33.91 (−)
Core-shell	34.13 (+)	24.83 (−)

It seems that an increase in fiber diameter and consequently a reduction in pore size resulted after 28 weeks of degradation for all scaffolds. The greatest increase in fiber diameter was observed in the tri-layered scaffolds (33.91%) in which the reduction in pore size was the highest. This was followed by core-shell scaffolds with a pore size reduction amount of 24.83% and the single-layered with 16.14%

### Mechanical properties of the scaffolds

**Fig. 9:**
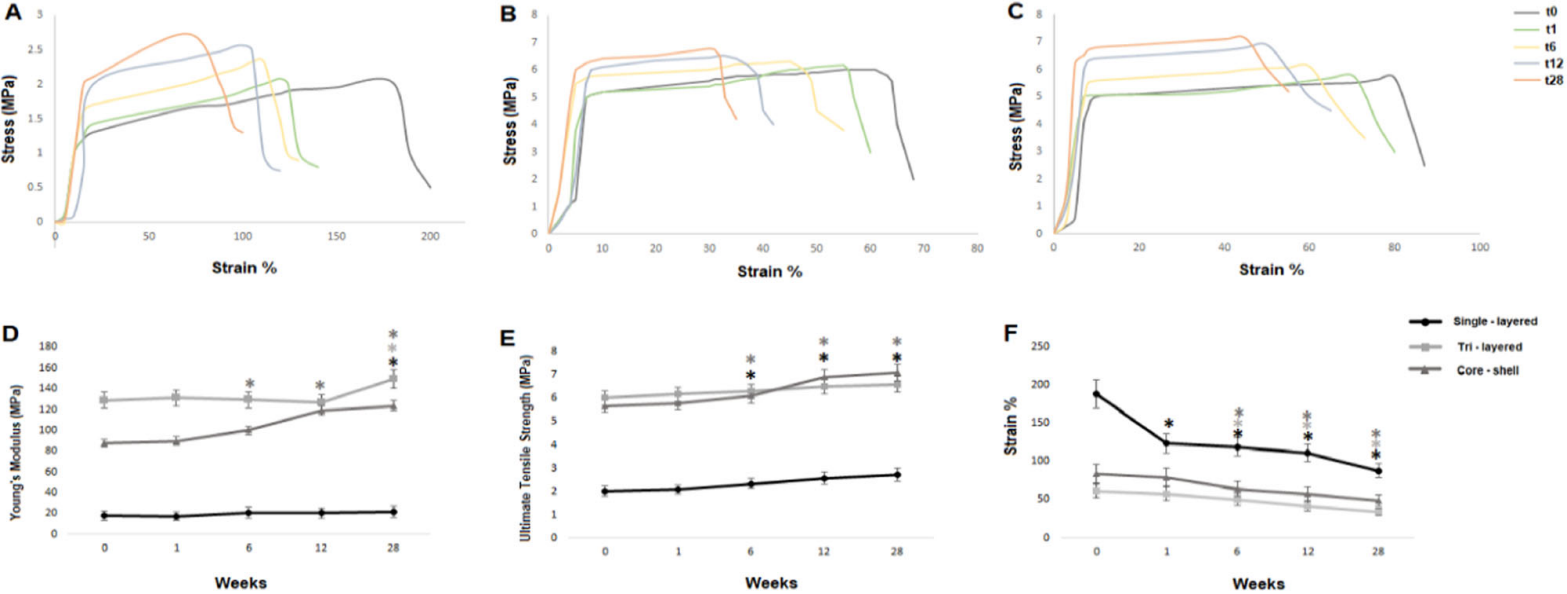
Mechanical properties of the scaffolds following hydrolytic degradation in phosphate buffered saline solution (PBS) at 37 °C for 28 weeks: **A**, **B** and **C** stress-strain curves for single-layered, tri-layered and core-shell electrospun scaffolds. Tensile properties of single-layered, tri-layered and core-shell electrospun fibers: **D** Young’s modulus, **E** ultimate tensile strength and **F** percentage strain at failure. (mean ± SD, *n* = 5), *p < 0.05. Stars (*) represent statistical significance compared to non-degraded scaffold (time zero)

A TE scaffold should possess suitable mechanical properties to withstand the physiological mechanical forces experienced by the regenerated tissue. However, as the scaffold material undergoes in vivo biodegradation, its initial mechanical strength gradually diminishes. It is crucial to assess and match this decrease with the mechanical strength of the newly formed tissue. In this study, we investigated the Young’s modulus, ultimate mechanical strength, and strain at failure throughout the entire degradation period to determine the extent of the drop in mechanical properties. The stress-strain curves for each scaffold type, both before the initiation of hydrolytic degradation (time zero) and during the degradation process (1, 6, 12 and 28 weeks), are illustrated in Fig. 9. Specifically, Fig. 9A depicts the mechanical behavior of single-layered scaffolds, Fig. 9B captures the mechanical response of tri-layered scaffolds, and Fig. 9C similarly focuses on the mechanical characteristics of core-shell scaffolds. These curves provide a thorough overview of the mechanical evolution from time zero (t0) to 28 weeks (t28) under hydrolytic degradation in PBS for each scaffold type.

#### Young’s Modulus

More specifically, Fig. 9D shows the Young’s modulus after tensile mechanical testing of the three scaffolds. Single-layered PCL scaffolds at time zero demonstrated the lowest modulus (18 ± 4.5 MPa) compared to core-shell (88 ± 5.1 MPa) and tri-layered (129.2 ± 11.8 MPa) scaffolds. During the degradation process Young’s modulus increased significantly for single-layered scaffolds (18%) after 28 weeks, reaching a final value of 21.24 ± 5.31 MPa. For tri-layered scaffolds, a significant increase in Young’s modulus (15.63%) was also observed, rising to 149.4 ± 13.64 MPa at the final time point. Core-shell scaffolds presented a similar trend, with a sharp increase (40.7%) measured over time, to a final mean modulus of 123.8 ± 4.95 MPa.

#### Ultimate Tensile Strength

Figure 9E shows the ultimate tensile strength of the tested groups. It seems from the results that a similar trend to the Young’s modulus was followed during degradation, where an overall increase in strength of all the three groups was achieved after 28 weeks. The single-layered samples (time zero) demonstrated the lowest strength value (2.02 ± 0.18 MPa), followed by the core-shell scaffolds (5.68 ± 0.28 MPa) and the tri-layered scaffolds (6.13 ± 0.31 MPa). A gradual increase for single-layered degraded samples was observed over time, being 2.97%, 15.84%, 27.29% and 35.15% higher after 1, 6, 12 and 28 weeks (2.08 ± 0.21 MPa, 2.34 ± 0.23 MPa, 2.57 ± 0.26 MPa and 2.73 ± 0.27 MPa, respectively). For tri-layered samples after 1 week a 2.66% (6.18 ± 0.31 MPa) increase in strength was observed, followed by a 4.67%, 8.31% and 12.96% rise after 6, 12 and 28 weeks (6.3 ± 0.32 MPa, 6.52 ± 0.43 MPa and 6.8 ± 0.36 MPa, respectively). For the core-shell samples, a similar trend to tri-layered scaffolds was observed. Specifically, after 1 week a 1.94% (5.79 ± 0.28 MPa) increase in strength was observed, followed by a 7.75%, 21.48% and 25.35% rise after 6, 12 and 28 weeks (6.12 ± 0.31 MPa, 6.9 ± 0.35 MPa and 7.12 ± 0.36 MPa, respectively).

#### Maximum Strain

Figure 9F shows the maximum strain at failure of the scaffolds stored in PBS for up to 28 weeks. The greatest strain (189 ± 22.3%) was observed for single-layered scaffolds at time zero (week 0), which became less ductile over time, with maximum strain at break being 88 ± 11.4% (28 weeks). The most brittle of the three groups, tri-layered scaffolds demonstrated the lowest strain overall, with a decrease from a strain of 61 ± 6.22% to a final strain of 34 ± 5.1% after storage for 28 weeks. The core-shell scaffolds also demonstrated a reduction from 84 ± 12.6% (time zero) reaching 49 ± 7.35% after 28 weeks of storage in PBS.

### Biocompatibility of scaffolds

**Fig. 10:**
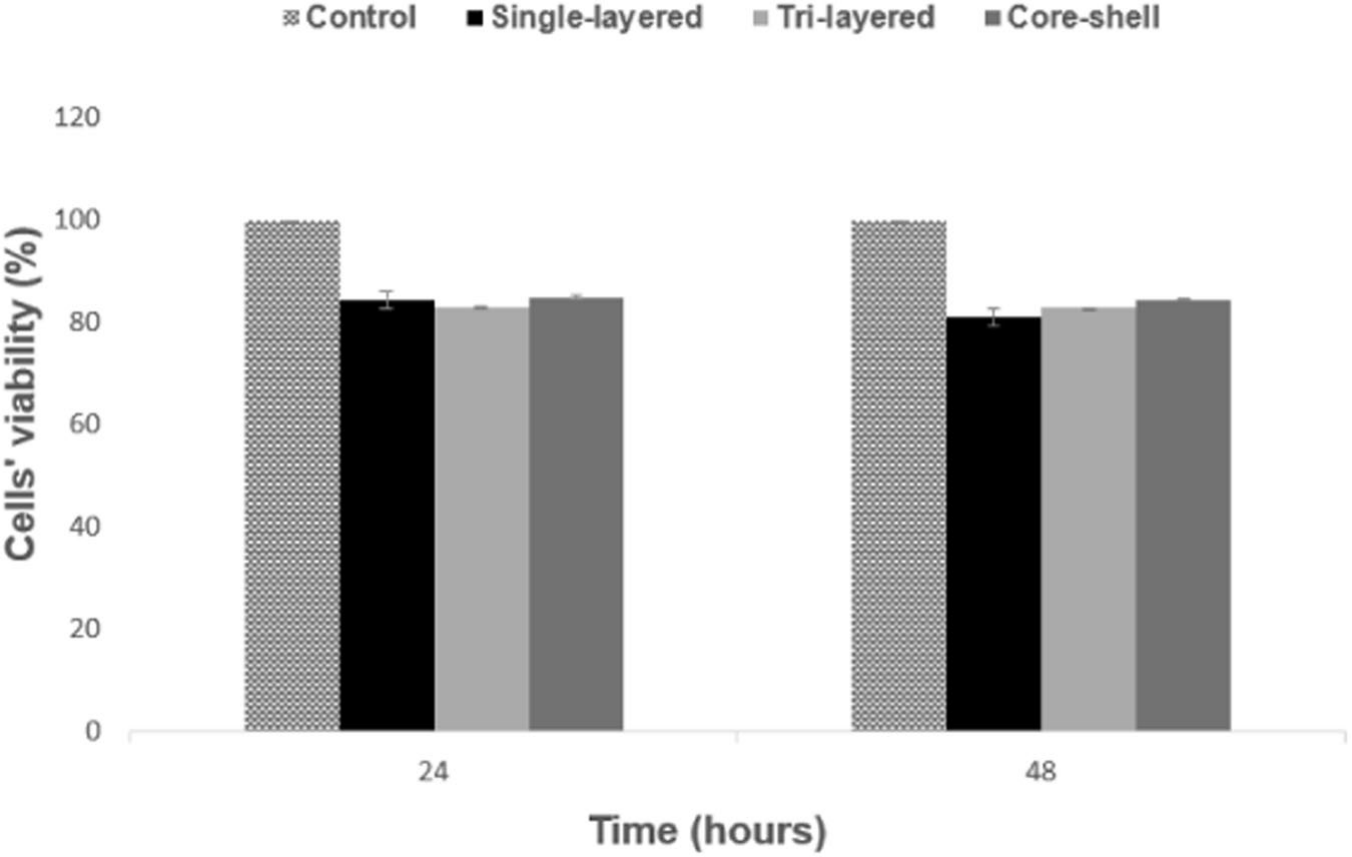
Viability by MTT assay of HEK-293 cells after 24 and 48 h of incubation with the three electrospun materials (single-layered, tri-layered, core-shell). Results were considered in comparison with the correspondent growth control (PS) at 24 or 48 h of incubation. (mean ± SD, *n* = 4)

Figure 10 depicts the MTT assay results for the quantitative evaluation of cytotoxicity of the electrospun materials. Our results showed that cytotoxicity was not observed, exhibiting high cell viability for all types of scaffolds after 48 hours. Concretely, after 24 hours of exposure, the tri-layered scaffolds presented the highest viability of HEK-293 cells with a percentage of 94.58%, followed by the core-shell scaffolds with a percentage of 92.95%, and finally the single-layered scaffolds exhibiting the lowest percentage of 92.41%. After 48 hours, for the core-shell scaffolds the percentage of cell viability remained high with a value of 91.92%. The single-layered scaffolds showed a decrease in the cell viability with a value of 88.61%, while the minimum cell viability observed was 87.59% for the tri-layered scaffolds after 48 hours of exposure.

Our results show no significant toxicity for the above electrospun materials, as they all exhibited sufficiently high percentages of cell viability after 48 hours, according to MTT assay. However, as a potentially better candidate biomaterial that can promote cellular effects appears to be the scaffold with a core-shell structure, since the cell viability rate remained high and almost invariant after 48 hours.

### Cell morphology, viability, and growth pattern

**Fig. 11:**
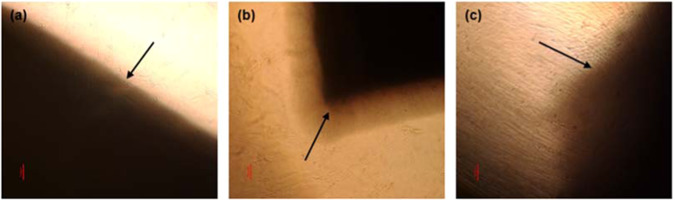
Inverted microscopy images of HEK-293 cells proliferation on the scaffolds after 7 days of culture **a** Single-layered scaffold **b** tri-layered scaffold and **c** core-shell scaffold. The dark areas of the images as shown by arrows are the scaffolds (scale bar = 100 μm)

**Fig. 12:**
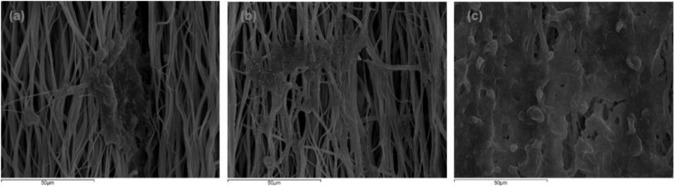
Representative SEM images of HEK-293 cells proliferation on the scaffolds after 7 days of culture **a** Single-layered scaffold **b** tri-layered scaffold and **c** core-shell scaffold (scale bar = 50 μm)

Inverted and SEM images of cultured HEK-293 cells on the electrospun fibrous scaffolds after 7 days of cell culture are shown in Figs. 11 and 12 respectively. The morphology and viability of the cells on the scaffolds’ fibers, as well as cells’ orientation towards them, were observed by an inverted microscope (Fig. 11). As can be observed, the cell-scaffold interaction was different for each type of scaffold. Cells were present in all scaffolds, but they seem to better attach, spread, and retain a spindle-like configuration on the core-shell scaffolds (Fig. 11c), exhibiting a higher cell integration and density in comparison with the poor performance of the single-layered PCL (Fig. 11a) and the tri-layered PVA-PCL-PVA (Fig. 11b) scaffolds.

Figure 12 shows the SEM micrographs of the interaction between cells and electrospun fibrous scaffolds after 7 days of cell culture. The results of the electron microscopy showed that in the single-layered scaffolds, cells appeared to infiltrate and aggregate between the fibers of the scaffold. Alike, cells in the tri-layered scaffolds seemed to attach to the aligned fibers, with a better spreading formation, but still low for the incubation period. On the other hand, the cells in the core-shell scaffolds presented a better proliferation rate as they were grown homogeneously on the fibers. The cells were not simply attached, but also integrated with the scaffold fibers confirming cellular infiltration. That led to the formation of a monolayer of HEK-293 cells that covered the entire scaffold surface (Fig. 12c), with cells displaying a high order cell distribution.

**Fig. 13:**
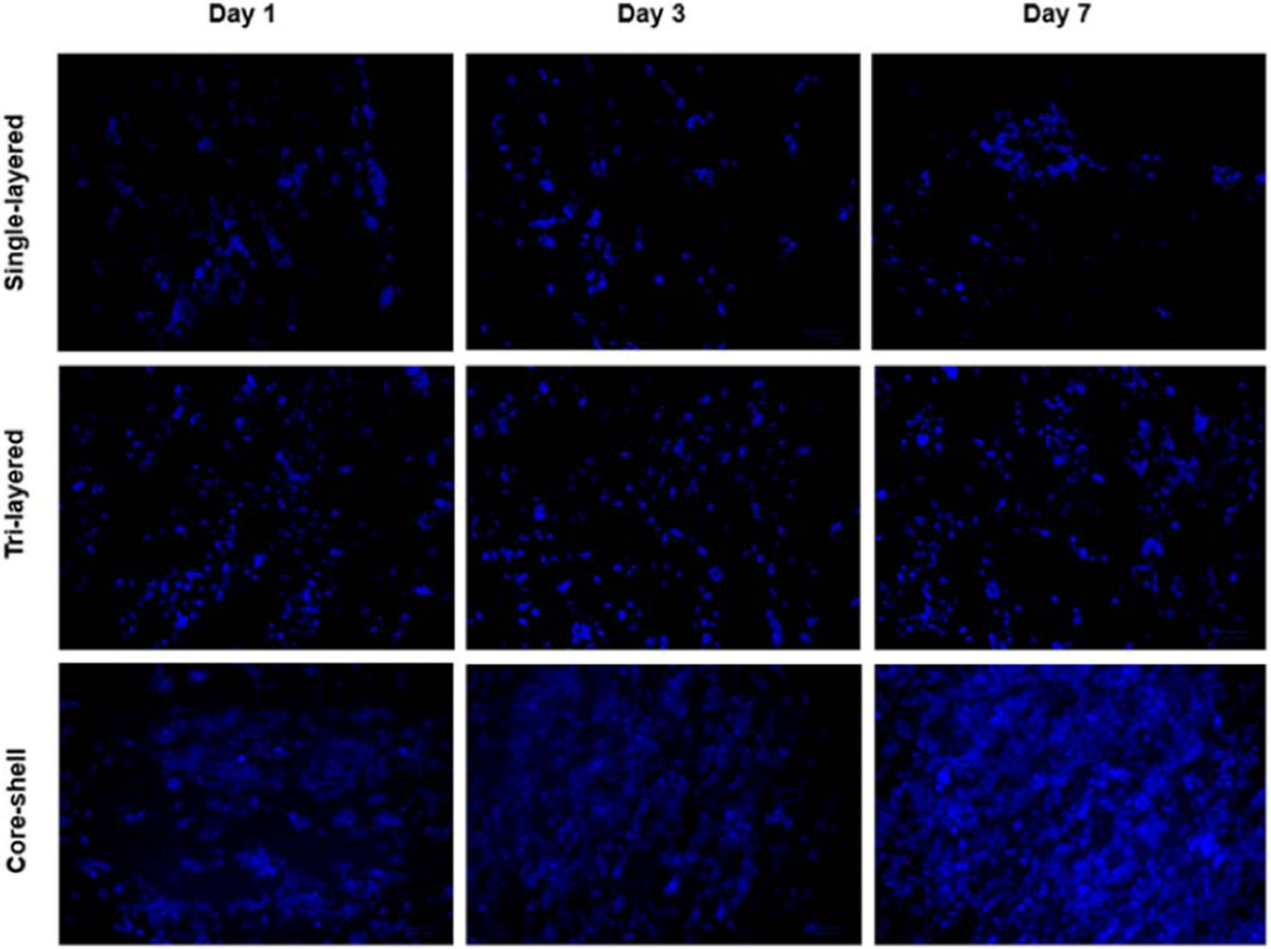
Fluorescent microscopic images of HEK-293 cells on the electrospun scaffolds on days 1, 3 and 7 of incubation. The nuclei of cells were stained with DAPI (blue) (scale bar = 100 μm)

**Fig. 14:**
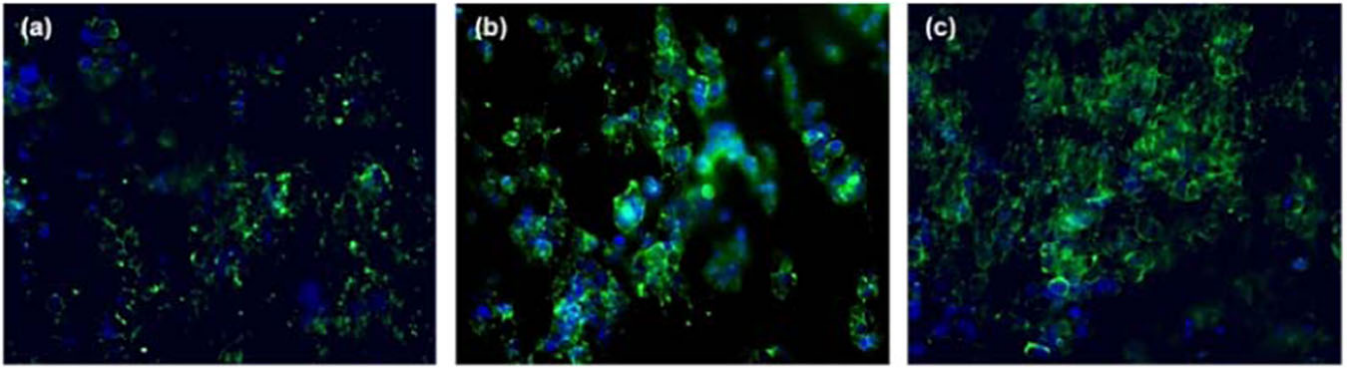
Fluorescent microscopy merge images of F-actin (Alexa Fluor® 488 Phalloidin, green) and cell nuclei (DAPI, blue) staining of HEK-293 cells after 7 days culture on the electrospun fibrous scaffolds **a** Single-layered scaffold **b** tri-layered scaffold and **c** core-shell scaffold (scale bar = 100 μm)

HEK-293 cells were also fixed and stained with DAPI and Alexa Fluor® 488 Phalloidin, as described in the experimental section, to visualize the nucleus and the actin filaments respectively, as seen in Figs. 13 and 14. A ZOE Fluorescent Cell Imager (Biorad) was used to visualize the cells up to 7 days of growth on the electrospun scaffolds. After 1 day of incubation, it can be observed (Fig. 13) that the cells were well attached to all nanofibrous scaffolds, indicating cell adhesion, morphology, and growth pattern. As the cell culture days progressed, a gradual increase in the number of cells attached to all types of scaffolds was observed. However, after 7 days of incubation a tendency to increase the proliferation rate of the cells was only observed in the core-shell fibrous scaffolds. This result was further confirmed by fluorescence microscope images (Fig. 14), where merged images of HEK-293 cells stained for F-actin (Alexa Fluor® 488 Phalloidin, green) and cell nuclei (DAPI, blue) were captured after 7 days of culture on the electrospun scaffolds.

## Discussion

The study presented in this paper aimed to investigate the potential of the coaxial electrospinning technique for producing functional bioactive and cell-compatible fibrous TE scaffolds with tailored surface properties. The technique involves the use of a combination of hydrophilic and hydrophobic polymers co-processed in tri-layered and coaxial core-shell composite structures. Hydrophobic PCL polymer was selected due to its long-term functional stability in vivo and hydrophilic PVA due to its excellent biocompatibility [15, 20, 41, 42, 46]. The study involved the fabrication and comprehensive comparison of three different electrospun fibrous scaffolds: (1) pristine hydrophobic PCL structure as single-layer scaffolds, serving as a reference scaffold, (2) consecutive tri-layer scaffolds composed of hydrophilic PVA for external sides (PVA/PCL/PVA), and (3) core-shell scaffolds (PVA as shell and PCL as core). The scaffolds were characterized for their surface morphology, mechanical, and biological properties. Furthermore, a seven-month in vitro degradation study was performed on the electrospun scaffolds to evaluate alterations in their materials’ properties when exposed to simulated body fluids.

The choice of the appropriate set of the electrospinning parameters (such as voltage, feed rate, needle to collector distance, and rotary collector angular velocity) and the maintenance of optimal environmental conditions (including room temperature and humidity) were found to obtain a stable electrospinning process leading to fibers with a highly homogeneous size distribution and structure.

Visualizing the scaffolds under the scanning electron microscope revealed aligned fibers that overlapped forming multiple layers, leading to a very dense three-dimensional structure for the scaffolds, which is an indication of a successful electrospinning of the prepared solutions. As it can be observed, smooth, uniform and round-shaped fibers with an apparent aligned morphology and without beads or defects were produced (Fig. 2).

The characterization of the micro-nanotopography of the fibrous scaffolds is very important as it provides insights into the interconnectivity of the fibers and their potential to facilitate cellular infiltration, a crucial factor for in vivo tissue regeneration. The fibrous structure of electrospun scaffolds, which should imitate ECM, is essential for proper cell infiltration, attachment, proliferation, differentiation, and function. Both fiber diameter and pore size are crucial parameters in tissue regeneration applications. Notably, all the fabricated scaffolds in this study demonstrated nano- and micro-scale fiber diameters along with micro-scale pore sizes (Table 1). These findings indicate their suitability for tissue engineering applications. In particular, the achieved fibers diameters of the produced electrospun scaffolds are comparable to the collagen fibers found in native vessels [47], while their pore sizes are capable to permit cell infiltration through them [48]. In addition, according to the results of cell culture, it has been observed that the reduction in the diameter of the fibers of the scaffolds leads to a higher degree of cell proliferation and spreading, as well as a lower degree of cell aggregation, which is in accordance with the results of other studies [49–51]. Comparing the results of our study, the core-shell scaffolds demonstrate a significantly narrower diameter distribution, indicating a more uniform fiber structure.

The surface wettability of scaffolds is regarded as one of the desired material properties in tissue engineering and has a major impact on their biocompatibility and cell attachment [15, 20]. Generally, cells adhere to moderately hydrophilic surfaces; therefore, in scaffold fabrication, many surface modification techniques are applied to hydrophobic surfaces, such as plasma treatment [34, 52]. Based on existing literature and input from various material supply companies, a 50 °C.A. value for commercial tissue culture flask polystyrene (TCPS) is widely regarded as the optimal range for promoting cell adhesion [15, 35, 52]. Therefore, the moderately hydrophilic (C.A. 43^o^) core-shell structure obtained from coaxial electrospinning technique is highly conductive to cell attachment and proliferation, compared with the other two scaffolds (Fig. 4).

The results from in vitro degradation tests revealed a clear influence of the hydrophilic PVA component on the water uptake of the scaffolds. The combination of PVA with PCL significantly enhanced the scaffold’s ability to absorb water. This suggests that PVA may act as a wetting agent, facilitating the spread of water and hydrolysis products across the surface of the PCL layer in tri-layered scaffolds and the PCL core in core-shell scaffolds.

The weight loss tests conducted during accelerated degradation confirmed the presence of hydrophilic PVA in conjunction with the hydrophobic PCL in the scaffolds. The results indicated that a significant portion of the mass loss was attributed to hydrolysis mechanism(s), with PVA being more affected than PCL. A comparison between the tri-layered and core-shell scaffolds revealed that the tri-layered scaffolds experienced a gradual reduction in total thickness, primarily due to mass loss occurring at the two external PVA layers. In contrast, the core-shell scaffolds demonstrated minimal impact on the overall scaffold structure, as only the thin shell of the coaxial fibers was primarily affected. Consequently, the core-shell fibrous structure exhibited prolonged biodegradation, which could potentially eliminate limitations leading to reduced tissue attachment, proliferation, vascularization, and foreign body reaction [53]. These findings highlight the potential of core-shell scaffolds for tissue engineering applications.

From a macroscopic point of view, it seems that all scaffolds strongly retained external membranous surface morphology after the degradation process, indicating that the electrospun scaffolds could maintain their structural integrity for long duration (Fig. 7). Handleability of the tissue-engineered membranes after the initiation of the degradation process is an essential feature that facilitates their use and application during surgery [54]. Furthermore, a shrinkage of the total membrane dimensions of the scaffolds in the PBS solution under incubation conditions is minimized, a prerequisite step for in vitro cell culture studies.

A gradual thickening and flattening of the fibers of tri-layered and core-shell scaffolds was microscopically revealed after 6 and 12 weeks in PBS (Fig. 8). In contrast, the single-layered scaffolds exhibited relatively smooth fibers, albeit with noticeable areas of fused fibers. This may be associated with the presence of the products of polymer hydrolysis, especially of the PVA material, not yet fully decomposed and diluted to the degradation solution. After 28 weeks of incubation, the tri-layered and core-shell scaffolds displayed dense regions with evident signs of degradation, along with perpendicular fissures to the fiber direction. These regions were less pronounced in the single-layered scaffold at this stage. It is also worth noting that no changes in fiber alignment were observed throughout the degradation process for all types of scaffolds.

An essential characteristic of electrospun TE scaffolds is the morphology of their fibers and their postoperative behavior upon implantation in vivo. The way the fibers are connected and stacked together not only affects the structural integrity and mechanical properties of the scaffold; it can significantly affect how cells integrate and proliferate [55]. Due to the water absorption of polymers, an increase in fiber diameter was observed in all scaffolds. Even for hydrophobic single-layered scaffolds, fiber degradation was present, but occurred very slowly. This may be due, besides to their hydrophobic character, to the fact that these scaffolds after the electrospinning process were thicker (0.14 ± 0.01 mm) compared with the tri-layered (0.11 ± 0.02 mm) and core-shell (0.09 ± 0.01 mm) scaffolds [56]. The findings from Table 2 showed an increase in fiber diameters and a concurrent decrease in pore size after 28 weeks of degradation. These results align with the observations made through SEM, which indicated a higher degradation rate of the PVA content within the fibers.

An overall increase in scaffold stiffness (Young’s modulus and ultimate tensile strength) was obtained (Fig. 9D, E) for all degraded samples compared to time zero (before degradation process) following storage in PBS (37 °C), being significantly different for tri-layered and core-shell scaffolds. This can be attributed to the absorption of water by the scaffolds, particularly the PVA compartment, leading to swelling, increased fiber diameter, and reduced pore size. Besides that, the presence of undiluted crystalline hydrolysis products between the fibers likely contributed to the increased scaffold stiffness [57]. This was also verified by the decrease in maximum strain that scaffolds withstand before failure, as shown in Fig. 9F, indicating decrease in ductile properties of polymers due to increased crystallinity. However, the hydrolysis of polymers in PBS is a complicated mechanism, involving among others the breakage of side chains or even backbone chains, fracture of intermolecular crosslink bonds etc., depending on the type of polymer [58]. A comprehensive investigation of the degradation procedure necessitates the utilization of additional techniques and procedures, which fall beyond the scope of this study.

The cell culture studies revealed notable differences in cell compatibility among the three examined scaffold types. The limited growth and proliferation rate of HEK-293 cells in the single-layered scaffolds can be attributed to the low hydrophilicity of PCL. As previously discussed, the hydrophilic-hydrophobic characteristics of a scaffold play a crucial role in tissue culture, significantly influencing initial cell adhesion and migration [15, 20, 34]. One of the major obstacles of electrospun scaffolds fabricated with synthetic polymers such as PCL is low cell affinity due to low hydrophilicity and lack of surface cell-recognition sites. Indeed, the previous results of contact angle measurements of single-layered fibrous scaffolds gave a C.A. of 119° (Fig. 4a), indicating that these scaffolds are highly hydrophobic. However, the incorporation of a hydrophilic polymer, such as PVA, in combination with the hydrophobic PCL, led to a significant improvement in the hydrophilicity of these scaffold types, resulting in a more favorable cellular response, as confirmed by the images in Figs. 13 and 14.

Overall, from MTT assay data and the microscopic image analysis, it can be concluded that the core-shell electrospun fibrous scaffolds exhibited remarkable cell compatibility, effectively promoting cell adhesion and proliferation. Furthermore, the cells demonstrated a distinctive alignment and a well-defined orientation on the core-shell scaffolds, setting them apart from the single-layered and tri-layered scaffolds.

## Conclusions

This study aimed to develop novel tissue engineering (TE) scaffolds by combining hydrophobic polymers, such as PCL, exploiting its in vivo mechanical durability, with hydrophilic polymers like PVA, which degrade rapidly but offer enhanced biocompatibility. The findings from a comprehensive range of morphological, physicochemical, mechanical, and biological tests conducted in this study consistently demonstrated that the core-shell scaffolds, comprising a combination of PCL (core) and PVA (shell), exhibited exceptional promise for applications in tissue engineering (TE). The fabricated scaffolds effectively synergized the advantageous characteristics and properties of both polymers, namely the exceptional mechanical strength and ductility of PCL, alongside the desirable bioactivity and hydrophilicity inherent in PVA. They were able to balance their degradation rate and provide favorable surfaces for cell growth and proliferation, even without external stimuli or growth factors. Comparatively, the tri-layered PVA/PCL/PVA scaffolds, while offering favorable cell-friendly interaction, exhibited sensitivity to rapid degradation and relatively poor mechanical properties.

The study demonstrated the potential for the coaxial electrospinning technique to produce functional bioactive and cell-compatible fibrous scaffolds with tailored surface properties. The core-shell scaffolds developed in this study could provide an attractive option for TE applications based on their unique architecture, which degrades smoothly, avoids burst effects, maintains good long-term mechanical properties, and supports cell growth. The results of this study also highlight the importance of continued research in the field, including further cellular studies using mesenchymal stem cells (MSCs), before proceeding to animal studies to evaluate in vivo cell-scaffold interactions.
